# Using diffusion tensor imaging to detect cortical changes in fronto-temporal dementia subtypes

**DOI:** 10.1038/s41598-020-68118-8

**Published:** 2020-07-08

**Authors:** M. Torso, M. Bozzali, M. Cercignani, M. Jenkinson, S. A. Chance

**Affiliations:** 1 Nuffield Department of Clinical Neurosciences, University of Oxford, Oxford, UK; 2 Oxford Brain Diagnostics, Oxford Centre for Innovation, New Road, Oxford, OX1 1BY UK; 3 Neuroimaging Laboratory, Santa Lucia Foundation, Rome, Italy; 4 ‘Rita Levi Montalcini’ Department of Neuroscience, University of Turin, Turin, Italy; 5 Clinical Imaging Sciences Centre, Department of Neuroscience, Brighton and Sussex Medical School, University of Sussex, Brighton, UK; 6 Wellcome Centre for Integrative Neuroimaging, FMRIB, Nuffield Department of Clinical Neurosciences, University of Oxford, Oxford, UK

**Keywords:** Dementia, Cognitive ageing

## Abstract

Fronto-temporal dementia (FTD) is a common type of presenile dementia, characterized by a heterogeneous clinical presentation that includes three main subtypes: behavioural-variant FTD, non-fluent/agrammatic variant primary progressive aphasia and semantic variant PPA. To better understand the FTD subtypes and develop more specific treatments, correct diagnosis is essential. This study aimed to test the discrimination power of a novel set of cortical Diffusion Tensor Imaging measures (DTI), on FTD subtypes. A total of 96 subjects with FTD and 84 healthy subjects (HS) were included in the study. A “selection cohort” was used to determine the set of features (measurements) and to use them to select the “best” machine learning classifier from a range of seven main models. The selected classifier was trained on a “training cohort” and tested on a third cohort (“test cohort”). The classifier was used to assess the classification power for binary (HS vs. FTD), and multiclass (HS and FTD subtypes) classification problems. In the binary classification, one of the new DTI features obtained the highest accuracy (85%) as a single feature, and when it was combined with other DTI features and two other common clinical measures (grey matter fraction and MMSE), obtained an accuracy of 88%. The new DTI features can distinguish between HS and FTD subgroups with an accuracy of 76%. These results suggest that DTI measures could support differential diagnosis in a clinical setting, potentially improve efficacy of new innovative drug treatments through effective patient selection, stratification and measurement of outcomes.

## Introduction

Fronto-temporal dementia (FTD) is one of the most common types of presenile (< 65 years) dementia^1^, characterized by a heterogeneous clinical presentation that typically includes three main subtypes: behavioural variant (BV), semantic variant (SV) and primary progressive aphasias. A correct diagnosis is important to better understand the different subtypes and to develop more personalized treatments. Neuropathologically, patients with FTD show relatively selective frontal and temporal lobar degeneration (FTLD) characterized by atrophy, gliosis in atrophic cortices, and protein deposition forming distinct inclusion bodies in brain cells^2^.

Over the last decade, the continuing advances in neuroimaging have provided new opportunities to study the pathophysiological mechanisms of neurological diseases and to help in diagnosis. Structural MRI and CT show patterns of atrophy mainly in the fronto-temporal regions. Fluorodeoxyglucose positron emission tomography (FDG-PET), functional MRI, and single-photon-emission CT likewise show disproportionate hypoperfusion and hypometabolism in these regions^3^.

Some studies have suggested Tau imaging is a promising method with potential for further differentiating between Alzheimer’s disease, non-Alzheimer’s tauopathies, and tau-negative dementias^3,4^, although results are still contrasting^5,6^. Research in molecular PET imaging is very active, not only because of the specificity it allows for differentiation of fronto-temporal dementia from Alzheimer’s disease, but also because of its potential for further differentiating among frontotemporal lobar degeneration syndromes. However, the promising tau tracers require further development of novel compounds to detect different tau isoforms^7^. Detection of proteins using cerebrospinal fluid (CSF) biomarkers, instead of imaging methods, has potential to aid differential diagnosis between AD and FTLD, although it is an invasive method that still needs further investigation^8^.

An alternative to protein quantification is to further investigate the anatomy. While FTD is characterized by assessment of cortical atrophy, this is a relatively gross effect in neuropathological terms. Previous studies have suggested that the cellular organization in the cerebral cortex could be used as a potential biomarker of cortical damage in dementia^9,10^. For example, histological studies^9,10^ showed that changes in cortical architecture, caused by neurodegenerative processes and protein deposition, produced alteration in the cortical geometrical properties including disruption of minicolumnar cellular organisation. Minicolumn degeneration varies between brain regions, reflecting the typical pattern of tau tangle accumulation^11^. These differences between brain regions suggest that microstructural changes in cortical grey matter could be sensitive for differentiating between dementia variants. Some of these cytoarchitectural changes have been found to be correlated with measurements from analysis of neuroimaging data based on Diffusion Tensor Imaging (DTI) in the cortical grey matter^12^. DTI can show widespread white-matter degeneration in frontotemporal dementia, exceeding that seen in Alzheimer’s disease^13^, but until now, relatively little attention has been paid to the use of DTI to examine diffusion proprieties in grey matter structures. The sensitivity of DTI to changes in microstructural properties suggests that DTI may be a useful modality to detect correlates of, or perhaps even the precursors of, macroscopic atrophy.

In this study, we aimed to test some novel Diffusion Tensor Imaging (DTI) measures that had been previously validated in an ex-vivo comparison with post-mortem histology^12^. In the current study those measures were applied to in vivo scans in FTD patients based on the hypothesis that they may reflect cytoarchitectural changes in the cortex in FTD patients compared with a control group. We looked also for differences in the pattern of cortical diffusivity changes between FTD subtypes. Machine learning has been used previously to try to improve dementia diagnosis^14,15^. Therefore we investigated the use of a machine learning approach to test the discrimination power of these new DTI measures.

## Method

### Participants

A total of 96 subjects with probable FTD and 84 healthy subjects (HS) were included in the study.

The frontotemporal lobar degeneration neuroimaging initiative (FTLDNI) dataset was used to select subjects’ scans for the “selection cohort” and “test cohort” (Table 1). FTLDNI was founded through the National Institute of Aging and started in 2010. The primary aims of FTLDNI are to identify neuroimaging modalities and methods of analysis for tracking frontotemporal lobar degeneration (FTLD) and to compare the value of neuroimaging with other biomarkers in diagnostic roles. The Principal Investigator of FTLDNI is Dr. Howard Rosen, (University of California, San Francisco). The data is the result of collaborative efforts at three different sites in North America. For more information, please visit: https://memory.ucsf.edu/research/studies/nifd [https://ida.loni.usc.edu/collaboration/access/appLicense.jsp]. Access to the FTLDNI data was approved by the data access committee.

**Table 1:** Demographic and clinical characteristics.

Dataset	Diagnosis	Age [years]	Gender (F/M) [%]	Education [years]	MMSE Score [range]	CDR
Selection cohort (NIFD)	HS n = 30	68.3 ± 5.51	15/15	13.9 ± 3.10	28.8 ± 1.21 #	0 ± 0 #
	FTD n = 30	68.5 ± 6.07^a^	15/15^b^	13.4 ± 2.96^a^	21.2 ± 5.96^a^	0.74 ± 0.46^a^
Training cohort (Rome)	HS n = 30	67.2 ± 6.37	16/14	13.0 ± 3.01	28.9 ± 1.72 #	0 ± 0 #
	FTD n = 24	66.2 ± 5.11^a^	15/9^b^	10.9 ± 4.96^a^	21.9 ± 5.63^a^	0.72 ± 0.48^a^
Test cohort (NIFD)	HS n = 24	66.9 ± 5.84	14/10	16.2 ± 1.56	29.5 ± 0.72 #	0 ± 0 #
	FTD n = 42	67.5 ± 8.14^a^	16/26^b^	16.01 ± 3.09^a^	19.6 ± 5.68^a^	0.79 ± 0.51^a^

^a^t-test. ^b^Chi-square. HS: healthy subjects; FTD: Fronto-Temporal Dementia. MMSE: Mini Mental State Examination; CDR: Clinical Dementia Rating scale. For each group of subjects. The table shows the mean (SD) of age, years of formal education, MMSE and CDR scores and percentages of gender distribution. p < 0.05 after FDR correction. # Significant difference.

In order to avoid potential bias due to differences in acquisition parameters for B_0_ and DWI images, just the subjects with comparable acquisition protocol were selected. A balanced cohort of 30 FTD patients (10 bvFTD, 10 svPPA and 10 nfvPPA) and 30 HS was included in the “selection cohort”. The remaining subjects, 42 early FTD patients (15 bvFTD, 18 svPPA and 9 nfvPPA) and 24 HS were included in the “test cohort”.

The group of scans acquired in the Neuroimaging Laboratory of Santa Lucia Foundation in Rome was used as a "Training Cohort" (Table 1, inserted between “selection” and “test”) and included 24 FTD patients (5 bvFTD, 13 svPPA, 6 nfvPPA) and 30 HS.

All subjects underwent an extensive clinical and neuropsychological evaluation and an MRI scan. The diagnosis of FTD was made according to the current criteria^16,17^. Patients with vascular, psychiatric or other neurological disorders were excluded.

### MRI data acquisition and pre-processing

For the Selection Cohort and the Test Cohort, MR images were acquired on a 3 T Siemens Trio Tim system equipped with a 12-channel head coil at the UCSF Neuroscience Imaging Center, including the following acquisition: (1) T1 MPRAGE (TR/TE = 2,300/2.9 ms, matrix = 240 × 256 × 160, isotropic voxels 1 mm^3^, slice thickness = 1 mm); (2) Diffusion sequences were acquired using the following parameters: TR/TE 8,200/86 ms; , b factor = 2000s/mm^2^, isotropic voxels 2.2 mm^3^) this sequence collects 1 image with no diffusion weighting (b0) and 64 images with diffusion gradient applied in 64 non-collinear directions.

The Training Cohort scanning was performed at the Neuroimaging Laboratory of Santa Lucia Foundation in Rome using a 3 T Magnetom Allegra MRI scanner (Siemens Healthcare, Erlangen, Germany) operated with a 12-channel head coil, including the following acquisitions: (1) MDEFT (TR/TE = 1,338/2.4 ms, matrix = 256 × 224 × 176, resolution = 1 × 1 × 1 mm^3^, slice thickness = 1 mm); (2) diffusion-weighted (DW) twice-refocused spin echo echo-planar imaging (TR/TE = 10,200/85 ms, b factor = 1000 s/mm^2^, isotropic voxels 2.3 mm^3^), this sequence collects 7 images with no diffusion weighting (b0) and 61 images with diffusion gradients applied in 61 non-collinear directions.

The 3D T1-weighted image for each subject, was segmented using the recon-all script included in Freesurfer v 6.0 (https://surfer.nmr.mgh.harvard.edu/).

The segmented masks obtained were used to estimate the volumes of cortical and subcortical grey matter, total white matter, brain stem, corpus callosum, left and right hippocampus, left and right thalamus, left and right caudate, left and right putamen, left and right pallidum, left and right amygdala and left and right accumbens. To account for head size, all volumes were normalised for total intracranial volume and expressed as fractions.

All DTI images were processed using the FMRIB software library, (FSL Version 5.0.9, FMRIB, Oxford, UK, https://www.fmrib.ox.ac.uk/fsl/). Data was corrected for eddy currents and head movement and the diffusion tensor model at each voxel was fitted using DTIFIT.

To control for the effect of head movement^18^ in DTI maps, a displacement index generated using an in-house script was calculated. This index measured the absolute displacement of the head from one volume to the next and was calculated as the average of the absolute values of the differentiated realignment estimates obtained from eddy correction. This value was used as a covariate in the GLM multivariate analysis.

### Cortical Diffusivity analysis

Cortical Diffusivity analysis was performed using an in-house novel software tool. The tool generates cortical profiles, i.e. lines within the cortex in the vertical direction based on the columnar organisation of the cortex. Values for the diffusion tensor derived metrics were averaged along the cortical profiles, within the cortical grey matter^12^. The metrics calculated were MD, FA and three measures relating to the principal diffusion component^12^, namely: the angle between the cortical profile and the principal diffusion direction (AngleR); the principal diffusion component projected onto the plane perpendicular to the cortical profile (PerpPD, (× 10–3 mm^2^/sec)) and the principal diffusion component projected onto the cortical profile (ParlPD, (× 10–3 mm^2^/sec). All of the cortical values were averaged to reduce the influence of noise in the DTI scans, effectively smoothing the data, and ensuring only directionality with some local coherence would dominate, guarding against the influence of random deflections from the radial direction. Previous work has found that measures of the cyto- and myelo-architecture are relatively stable within a cortical subregion^19^ indicating that it is valid to find an average value for that region. The whole-brain DTI maps were used to extract a single value for each cortical region segmented using the recon-all pipeline of the FreeSurfer V 6.0 software package (https://surfer.nmr.mgh.harvard.edu/). The cortical regions segmented (for each hemisphere) were: banks of the superior temporal sulcus, caudal anterior cingulate, caudal middle frontal, cuneus, entorhinal, fusiform, inferior parietal, inferior temporal, isthmus cingulate, lateral occipital, lateral orbitofrontal, lingual, medial orbitofrontal, middle temporal, parahippocampal, paracentral, pars opercularis, pars orbitalis, pars triangularis, pericalcarine, postcentral, posterior cingulate, precentral, precuneus, rostral anterior cingulate, rostral middle frontal, superior frontal, superior parietal, superior temporal, supramarginal, frontal pole, temporal pole, transverse temporal, insula.

### Design and statistical analysis

In the first part of the study, we compared the cortical diffusion measurements of patient and control groups in all cohorts separately and together. In the second part of the study, we tested the discrimination power of our new diffusion measures for classifying participants into two groups (patients and healthy subjects) and into FTD subtypes (semantic variant-svPPA, behavioural variant -bvFTD, non-fluent/agrammatic variant primary progressive aphasia -nfvPPA) using a machine learning algorithm.

Statistical analyses were performed using IBM SPSS Statistics version 25 (SPSS, Chicago, IL).

The multivariate General Linear Model of SPSS was used to assess the between-group differences in cortical diffusion measures and GM_fr in our cohorts, using the diagnosis as a fixed factor and head movement^20^, scanner and age as covariates.

T-test was used also to investigate age, education, MMSE and CDR between groups. To calculate statistical differences in gender, Chi-square analysis was used.

One-way ANOVA was used to compare regional values between FTD subtypes. All statistically significant results reported remained significant after false discovery rate correction (FDR < 0.05) ^21^.

### Feature selection, classifiers and classification accuracy

**Figure 1:**
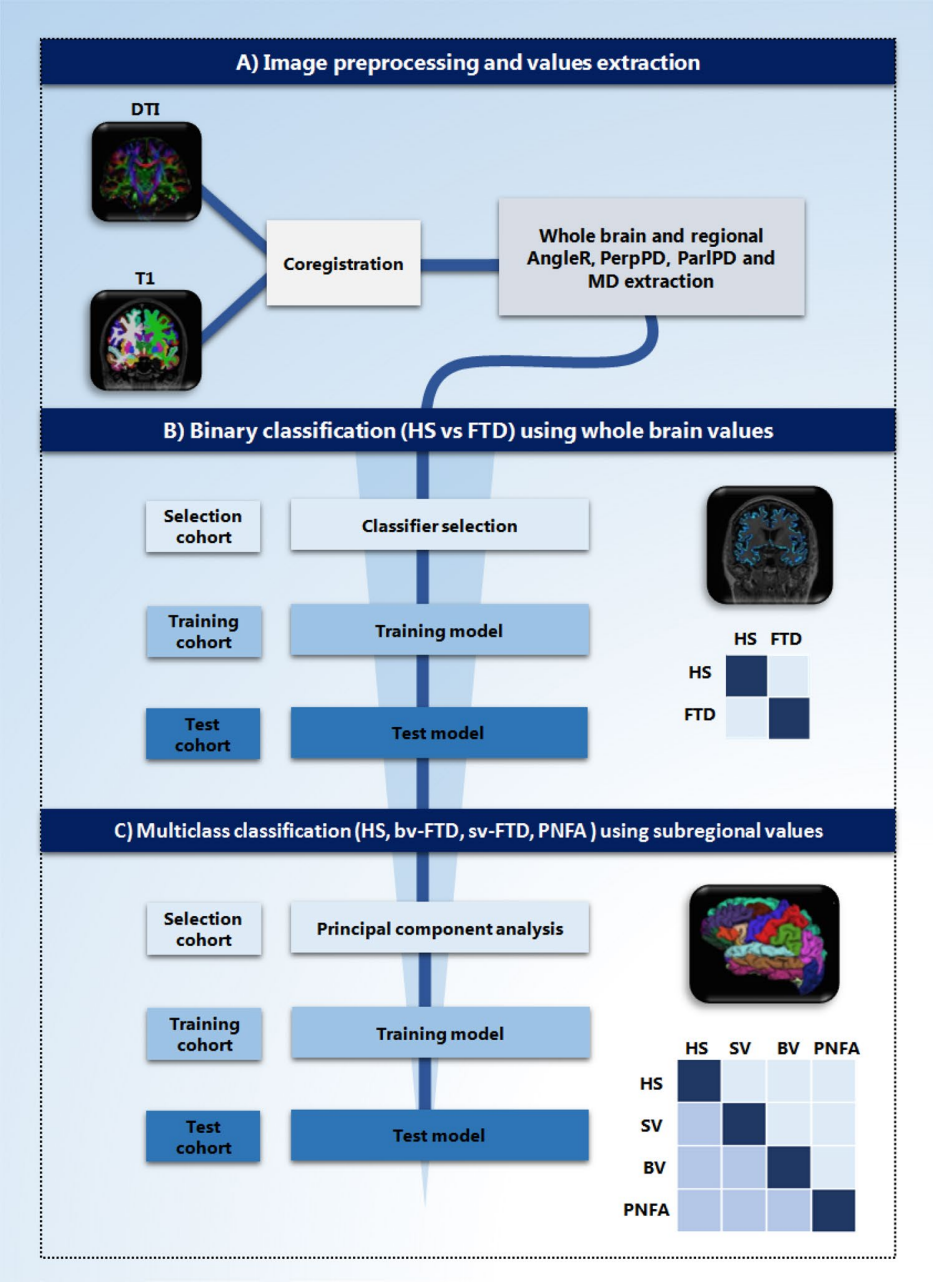
Overview of the study. (**A**) Image preprocessing and cortical diffusion measures extraction. (**B**) Binary classification using whole brain diffusion values. (**C**) Multiclass classification using subregional AngleR values. Both classifications were carried out including three different steps and cohorts: selection, training and test. (Image created using Microsoft Office Powerpoint 2010- www.microsoft.com).

To investigate the classification power of the DTI cortical measures to distinguish between patient and control groups and between the control group and FTD subgroups (bvFTD, svPPA and nfvPPA) several steps were required: (i) feature selection; (ii) identification of the best classification model from a set of plausible models using a “selection cohort”; (iii) training of the chosen classifier using the features selected on a training sample (training cohort); (iv) application of the classifier to an independent set (test cohort) that represented unseen data and provided an unbiassed test of accuracy (Fig. 1). In the binary classification all whole brain measures where used (AngleR, PerpPD, ParlPD, MD, GMfr and MMSE) while in the multiclass classification, the large number of initial features were reduced to improve the classification performance, removing irrelevant or redundant variables using principal component analysis (PCA) (SPSS Factor analysis) as a filter method on the “selection cohort”.

Many machine learning approaches have been trialed to classify subjects with dementia from elderly control subjects using a wide range of biomarkers^22–24^. In this study, a tenfold cross-validation scheme was used within the selection cohort to select the best classifier (evaluated on one fold and trained on the remainder) from a range of seven commonly used different supervised classification models: K-Nearest Neighbours (KNN), Support Vector Machine (SVM), ElasticNet (EN), Logistic Regression (LR), Random Forest classifier (RF), Gaussian NB (GNB) and Linear Discriminant Analysis (LDA). The best classifier was selected based on the majority vote from 1,000 runs of the cross-validation scheme, each using the same “best” features as calculated by principal component analysis in the selection cohort.

The classifiers were used to assess the classification power for both binary (HS vs FTD), and multiclass (HS vs bvFTD vs svPPA vs nfvPPA) classification problems.

The “best” model (the one with the highest accuracy) and features selected using the selection cohort, was trained on the training cohort. The final results reported are based on the performance in the test cohort.

In the binary classification all the features were used together and one at a time. All classification analyses were implemented in MATLAB 2018 (The Math).

The accuracy (ACC), sensitivity (SENS), specificity (SPEC), positive predictive value (PPV) and negative predicted value (NPV) were used to measure the discrimination performance.

To perform a more comprehensive classification among HS and the three clinical FTD subtypes, a multiclass classification was performed. This required a sub-regional analysis of all 68 brain regions. In order to avoid over testing, only the best cortical diffusivity measure was selected that had obtained the highest accuracy in the binary classification of global, whole brain data. This measure was then extracted from each brain region, for the 68 regional values in multiclass classification.

PCA analysis was applied to reduce the number of regional features in the selection cohort. The regional features selected in addition to the whole brain value, were used together in the classification.

The Accuracy (A), Sensitivity (SENS), Specificity (SPEC), positive predictive value (PPV) and the false discovery rate (FDR) were estimated to investigate the classification performance.

Finally, to investigate if the selected regional measures that were used as features in the multiclass classification were consistent with the pattern of cortical damage commonly described in the literature for each subgroup, a further one-way analysis of variance (ANOVA) was used to compare group differences in those regional values.

## Results

### Participants

Table 1 summarizes the principal demographic and clinical characteristics of all subjects who fulfilled the inclusion criteria, and thus entered the study.

In the selection cohort, no significant difference was observed between groups for age, years of formal education and gender. As expected, the t-test revealed between-groups differences in MMSE scores (*t* (58) = 5.979; *p* =  < 0.0001) and CDR (*t* (58) = − 6.460; *p* =  < 0.0001).

In the training cohort, no significant difference was observed between groups for age, years of formal education and gender. The t-test revealed higher MMSE (*t* (52) = 6.620; *p* =  < 0.0001) and CDR scores in the FTD group (*t* (52) =  − 8.195; *p* =  < 0.0001).

In the test cohort, no significant difference was observed between groups for age, years of formal education and gender but the FTD group showed significantly higher MMSE (*t* (64) = 5.016; *p* =  < 0.0001) and CDR scores (*t* (52) = − 6.865; *p* =  < 0.0001).

### Cortical diffusion and brain volumetric measurements

Multivariate GLMs were used to test for main effects of diagnostic group, with cortical measures (MD, AngleR, PerpPD, ParlPD and GMfr) as dependent variables, diagnostic group as the between-subjects factor (independent variable) and age and head movement as covariates.

In the Selection cohort the multivariate GLM showed significant effects of diagnostic group on cortical measures (*F*_5,55_ = 11.899; *p* =  < 0.0001). Age and head movement were not significantly associated with cortical measures and did not show interactions with diagnostic group.

The between-subjects effects for each cortical measures revealed a significant reduction of GMfr (*F*_1,59_ = 25.306; *p* =  < 0.0001) and increased MD (*F*_1,59_ = 18.151; *p* =  < 0.0001), AngleR (*F*_1,59_ = 41.151; *p* =  < 0.0001) and PerpPD (*F*_1,59_ = 23.153; *p* =  < 0.0001) values in FTD group compared to HS.

In the Training cohort the multivariate GLM showed significant effects of diagnostic group on cortical measures (*F*_5,49_ = 15.369; *p* =  < 0.0001). Age and head movement were not significantly associated with cortical measures and did not show interactions with diagnostic group.

The between-subjects effects for each cortical measures revealed a significant reduction of GMfr (*F*_1,53_ = 32.504; *p* =  < 0.0001) and increased MD (*F*_1,53_ = 25.877; *p* =  < 0.0001), AngleR (*F*_1,53_ = 36.808; *p* =  < 0.0001) and PerpPD (*F*_1,53_ = 18.959; *p* =  < 0.0001) values in FTD group compared to HS.

In the Test cohort the multivariate GLM showed significant effects of diagnostic group on cortical measures (*F*_5,61_ = 13.266; *p* =  < 0.0001). Age and head movement were not significantly associated with cortical measures and did not show interactions with diagnostic group.

The between-subjects effects for each cortical measures revealed a significant reduction of GMfr (*F*_1,64_ = 37.137; *p* =  < 0.0001) and increased MD (*F*_1,64_ = 22.933; *p* =  < 0.0001), AngleR (*F*_1,64_ = 33.041; *p* =  < 0.0001) and PerpPD (*F*_1,64_ = 36.574; *p* =  < 0.0001) values in FTD group compared to HS.

In another multivariate GLM, we compared all healthy subjects and FTD patients of all cohorts using cortical measures as dependent variables (MD, AngleR, PerpPD, ParlPD and GMfr) diagnosis as independent variables and age, movement and scanner as covariates. Results showed significant effects of diagnostic group (*F*_5,175_ = 40.912; *p* =  < 0.0001).

As in the previous analyses, the between-subjects effects for each cortical measures revealed a significant reduction of GMfr (*F*_1,179_ = 27.137; *p* =  < 0.0001) and increased MD (*F*_1,179_ = 26.962; *p* =  < 0.0001), AngleR (*F*_1,179_ = 57.868; *p* =  < 0.0001) and PerpPD (*F*_1,179_ = 55.705; *p* =  < 0.0001) values in FTD group compared to HS.

The analysis revealed also a significant effect of scanner model (*F*_5,175_ = 28.221; *p* =  < 0.0001) on MD (*F*_1,179_ = 9.127; *p* =  < 0.0001).

The healthy subjects groups from the three different cohorts were compared, using cortical measures as dependent variables (MD, AngleR, PerpPD, ParlPD and GMfr) cohort group as independent variables and age, movement and scanner as covariates.

The analysis revealed a significant effect of scanner model (*F*_5,79_ = 4.722; *p* =  < 0.0001) on MD (*F*_1,83_ = 2.325; *p* = 0.005). No other significant associations and interactions were detected.

Finally, the FTD group comparisons between the three cohorts, revealed just a significant effect of scanner model (*F*_5,91_ = 4.661; *p* =  < 0.0001) on MD (*F*_1,95_ = 2.285; *p* = 0.004).

Additional cortical and subcortical volumetric investigations were performed. (For more information see Supplemental).

### Feature selection and classifiers

Comparing the different classification models in the binary classification, our analysis of the selection cohort revealed that KNN was the best classifier (selected as the best in 96.6% of runs). We used KNN in both classification tasks (binary and multiclass classification).

Concerning binary diagnostic classification (HS vs FTD) all the whole brain features (MD, AngleR, PerpPD, ParlPD, GM_fr and MMSE) were used together and one at a time in the training cohort by the KNN classifier to train models, which were subsequently applied to the test cohort. The discrimination indices calculated in the test cohort were used to quantify the classification accuracy in that (Test) cohort and are summarized in Table 2. The model with all features selected by PCA had the highest classification accuracy (88%). When using the features independently, AngleR was the single feature with the highest accuracy (85%). Therefore, in order to avoid over-testing of a dataset of limited size, this best feature was used as the key measure in the multiclass classification. (See Fig. 1 for the analysis pathway).

**Table 2:** Accuracy indices of binary classification (HS vs FTD) in Test cohort.

Classifiers	Variables	N° of features	ACC %	SENS	SPEC	PPV	NPV
**KNN**	All features	6	88	0.85	0.90	0.89	0.86
	AngleR	1	85	0.95	0.67	0.83	0.89
	PerpPD	1	79	0.90	0.60	0.79	0.79
	PerpPD	1	77	0.80	0.75	0.76	0.79
	MD	1	72	0.70	0.75	0.74	0.71
	GM_fr	1	79	0.90	0.60	0.79	0.79
	MMSE	1	69	0.57	0.88	0.89	0.55

Accuracies for binary classification using whole brain measures (AngleR, PerpPD and Gm_fr) and MMSE. Abbreviations: *ACC* accuracy, *SENS* sensitivity, *SPEC* specificity, *PPV*  Positive predictive values, *NPV* negative predictive value.

To perform the multiclass classification (HS vs bvFTD vs svPPA vs nfvPPA), we carried out a PCA on the regional AngleR values in the selection cohort. The whole-brain AngleR value was also used as an additional feature. Table 3 shows a list of the 12 anatomical features selected (from a total of 68 regional features plus the single whole-brain feature) to perform the classification with the best classifier (KNN). The results on the test cohort revealed a classification accuracy of 76%. The confusion matrices, PPV, FDR, TP and FN percentages are shown in Table 4.

**Table 3:** Accuracy for multiclass classification (HS vs. svPPA vs bvFTD vs nfvPPA) in Test cohort.

Classifier	PCA features selected	N° of features	Accuracy %
**KNN**	AngleR whole brain	12	**75.75**
	AngleR caudalanteriorcingulate left		
	AngleR entorhinal left		
	AngleR fusiform left		
	AngleR parsopercularis left		
	AngleR precentral left		
	AngleR caudalmiddlefrontal right		
	AngleR inferiortemporal right		
	AngleR lingual right		
	AngleR precentral right		
	AngleR rostralmiddlefrontal right		
	AngleR temporalpole right		

Accuracies for multiclass classification of FTD subtypes.

**Table 4:** KNN multiclass confusion matrices of test cohort.

	HS	svPPA	bvFTD	nfvPPA		HS	svPPA	bvFTD	nfvPPA	SENS %	SPEC %
**HS**	**88.88** **(21)**	11.11 (2)	6,66 (1)	11.11 (1)	**HS**	**84**					**84**
**svPPA**	4,1 (1)	**72.22** **(13)**	13.33 (2)	11.11 (1)	**svPPA**		**76.47**			**76.47**	
**bvFTD**	4.1 (1)	11.1 (2)	**66.66** **(10)**	11.11 (1)	**bvFTD**			**71.42**		**71.42**	
**nfvPPA**	4.1 (1)	5.55 (1)	13.33 (2)	**66.66** **(6)**	**nfvPPA**				**60**	**60**	
**PPV %**	**88.88**	**72.22**	**66.66**	**66.66**							
**FDR%**	**11.12**	**27.78**	**33.34**	**33.34**							

**Figure 2:**
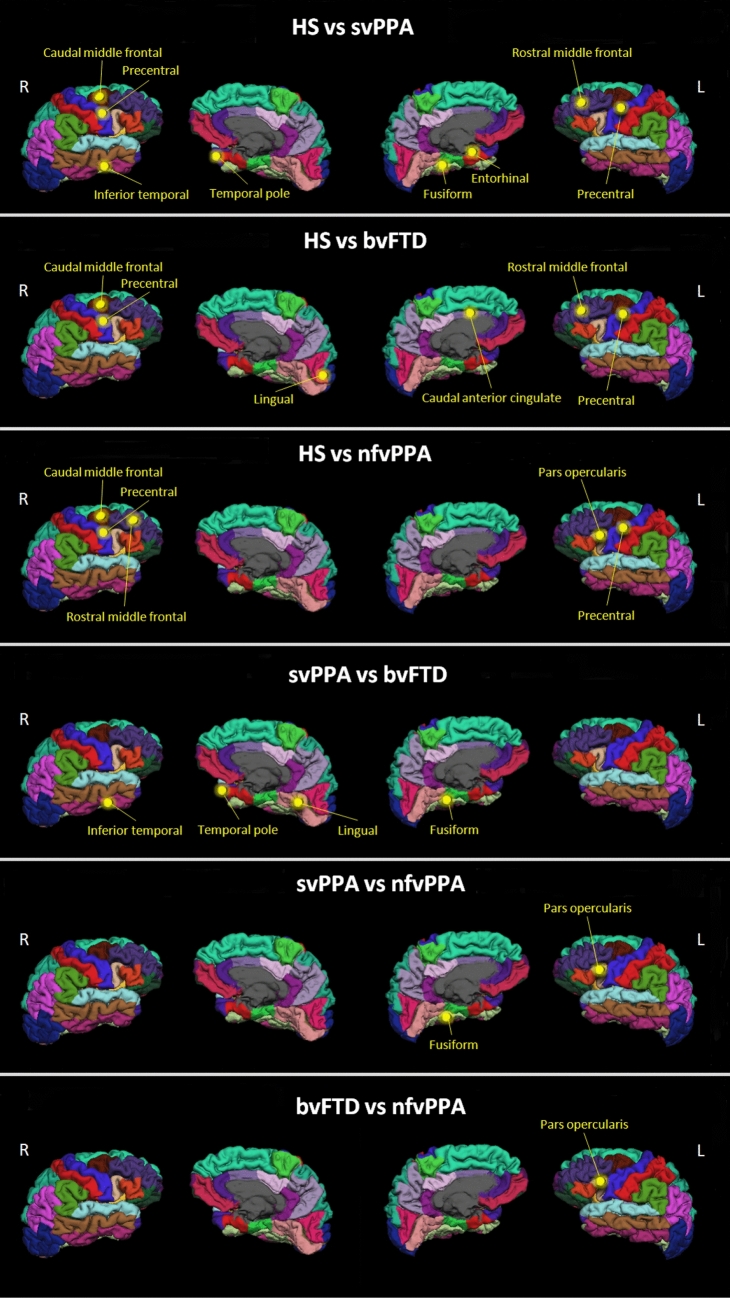
Multiclass classification. The regional AngleR values entered in the multiclass classification were used to compare FTD subgroups. The yellow dots indicate the cortical regions that were significantly different in post-hoc comparisons. (Image created using Microsoft Office Powerpoint 2010- www.microsoft.com).

The ANOVA post-hoc comparison results are summarized in Table 5 and Fig. 2. Compared with the HS group, all the other groups showed significant differences, mainly in frontal and temporal cortical regions.

**Table 5:** Post-hoc comparisons.

FTD subgroups	Features selected	vs HS	vs svPPA	vs bvFTD	vs nfvPPA
**svPPA**	AngleR caudalanteriorcingulate left	n.s		n.s	n.s
	AngleR entorhinal left	*p* = < 0.0001		n.s	n.s
	AngleR fusiform left	*p* = < 0.0001		*p* = < 0.0001	*p* = 0.002
	AngleR parsopercularis left	n.s		n.s	n.s
	AngleR precentral left	*p* = < 0.0001		n.s	n.s
	AngleR caudalmiddlefrontal right	*p* = < 0.0001		n.s	n.s
	AngleR inferiortemporal right	*p* = < 0.0001		*p* = 0.004	n.s
	AngleR lingual right	n.s		n.s	n.s
	AngleR precentral right	*p* = < 0.0001		n.s	n.s
	AngleR rostralmiddlefrontal right	*p* = < 0.0001		n.s	n.s
	AngleR temporalpole right	*p* = < 0.0001		*p* = 0.004	n.s
**bvFTD**	AngleR caudalanteriorcingulate left	*p* = < 0.0001	n.s		n.s
	AngleR entorhinal left	n.s	n.s		n.s
	AngleR fusiform left	n.s	n.s		n.s
	AngleR parsopercularis left	n.s	n.s		n.s
	AngleR precentral left	*p* = < 0.0001	n.s		n.s
	AngleR caudalmiddlefrontal right	*p* = < 0.0001	n.s		n.s
	AngleR inferiortemporal right	n.s	n.s		n.s
	AngleR lingual right	*p* = 0.001	*p* = 0.002		*p* = 0.002
	AngleR precentral right	*p* = < 0.0001	n.s		n.s
	AngleR rostralmiddlefrontal right	*p* = < 0.0001	n.s		n.s
	AngleR temporalpole right	n.s	n.s		n.s
**nfvPPA**	AngleR caudalanteriorcingulate left	n.s	n.s	n.s	
	AngleR entorhinal left	n.s	n.s	n.s	
	AngleR fusiform left	n.s	n.s	n.s	
	AngleR parsopercularis left	*p* = < 0.0001	*p* = < 0.0001	*p* = < 0.0001	
	AngleR precentral left	*p* = 0.0001	n.s	n.s	
	AngleR caudalmiddlefrontal right	*p* = 0.0001	n.s	n.s	
	AngleR inferiortemporal right	n.s	n.s	n.s	
	AngleR lingual right	n.s	n.s	n.s	
	AngleR precentral right	*p* = 0.0001	n.s	n.s	
	AngleR rostralmiddlefrontal right	*p* = 0.0001	n.s	n.s	
	AngleR temporalpole right				

One-way ANOVA. All *p* values reported remained statistically significant after false discovery rate correction (FDR < 0.05; 66 tests).

The analysis revealed that the svPPA group was more damaged than the bvFTD group in left fusiform (*F*_3,62_ = 15.374; *p* =  < 0.0001), right inferior temporal (*F*_3,62_ = 7.349; *p* = 0.004), right temporal pole (*F*_3,62_ = 8.281; *p* = 0.004) and in left fusiform cortex (*F*_3,62_ = 15.374; *p* = 0.002) compared with nfvPPA.

The bvFTD group appeared to have increased degeneration in right lingual cortex compared to the other two FTD groups (*F*_3,62_ = 12.176; bvFTD vs svPPA *p* =  < 0.0001; bvFTD vs nfvPPA *p* =  < 0.0001).

Finally, the nfvPPA group had more degeneration in left pars opercularis compared with the other two groups (*F*_3,62_ = 8.903; nfvPPA vs svPPA *p* =  < 0.0001; nfvPPA vs bvFTD *p* =  < 0.0001).

## Discussion

In the present study, we used a new set of whole-brain DTI measures, related to cortical microstructure, and a machine learning approach to distinguish normally aged healthy subjects from subjects with FTD in two independent cohorts. We also tested the differential diagnostic performance of our DTI measures to classify the different FTD subtypes on the basis of a set of regional cortical values.

The main findings of this work are: i) using six features (AngleR, PerpPD, ParlPD, MD, GM_fr and MMSE) the model was able to classify HS and FTD subjects with an accuracy of 88%; ii) using one of the new cortical DTI measures (AngleR) it was possible to classify HS and FTD subjects with an accuracy of 85%; iii) using a set of AngleR values from 12 cortical regions it was possible to obtain a differential diagnosis for all participants (HS, svPPA, bvFTD , nfvPPA) with an accuracy of 76%.

As shown in Table 2, the best HS vs FTD classifications were obtained using the novel cortical diffusion measures (AngleR, PerpPD and ParlPD), MD, GM_fr and the MMSE score, but a good classification was obtained also using just the AngleR value. This cortical diffusion measure, with the selected classifier (KNN) obtained the best performance as a single feature, compared with other cortical diffusion measures (PerpPD, ParlPD and MD) and with the GM_fr. We compared the performance of AngleR with GM_fr (widely used as an index of severity of GM atrophy), to test the relative merits of our DTI cortical measure. Indeed, GM atrophy is well-established as one of the main criteria for the diagnosis of neurodegenerative disorders. As shown in previous studies using histology^9–11^, the minicolumnar cytoarchitectural organization changes can be relatively independent from grey matter volumetric changes, especially in the early stages of neurodegenerative disorders. This independence is a possible explanation for why AngleR performs better than GM_fr, as the DTI measure might be sensitive to GM microstructural changes at an earlier stage than volumetric changes.

AngleR appeared to be sensitive to changes in neurodegeneration with a good accuracy. Therefore, AngleR and other cortical diffusion measures could be useful additions to the set of measures that are being tested to aid differential diagnosis and the early diagnosis of FTD.

Concerning the differential diagnosis of FTD subtypes, Table 3 shows the performance of the classifier using a set of features, selected by PCA, based on a number of AngleR values from different cortical areas. More specifically, we used the AngleR whole-brain values plus 11 out of 68 regional AngleR values.

Considering the small number of subjects in our cohorts, we decided not to ‘over-interrogate’ the data, instead focusing on the single feature that gave the best whole brain classification power—AngleR. In a larger study it could be possible to explore the sub-regional classification performance of other cortical diffusivity measures (e.g. PerpPD and ParlPD).

The performance of the classifier showed that using the selected set of features with the KNN classifier, an accuracy of 76% could be obtained for the differential diagnosis of the subjects into four different groups (HS, svPPA, bvFTD, nfvPPA). The classifier obtained a sensitivity of 84%, revealing a relatively high power to distinguish healthy subjects from FTD patients and therefore is encouraging if viewed in the light of the search for diagnostic screening power. However, the screening or diagnostic power of a test depends on threshold selection on the basis of a combination of sensitivity and specificity.

The confusion matrices (Table 4) describe the discrimination ability of the combination of whole-brain and regional AngleR values in classifying HS and subjects with an FTD subtype. The sensitivity (SENS) for each patient group shows that the selected features were able to classify more accurately svPPA patients (76%) with respect to patients with bvFTD (71%) and nfvPPA (60%). This difference could, in part, be due to the smaller number of samples in the Training and Test cohorts with nfvPPA diagnosis.

The cortical regions used in the multiclass classification correspond to those usually associated with FTD subtypes. To better understand the role of each regional value in the classification, we used the ANOVA post-hoc comparisons to identify the key regions for each group (Table 5). For the svPPA subtype our post-hoc comparisons showed that the main regions distinguishing svPPA and other groups were the left fusiform and entorhinal cortex, right temporal pole and right inferior temporal cortex. The left fusiform is one of the key regions involved in semantic tasks and can be particularly involved in semantic variant degeneration similar to the right temporal pole^25^, another brain region considered an important hub for semantic tasks^26^. In the svPPA group we also found higher values of AngleR in the right inferior temporal^27^ and the entorhinal cortex^28^.

The bvFTD group was characterized mainly by two cortical regions, left caudal anterior cingulate cortex and right lingual gyrus. As shown in previous studies, the left caudal anterior cingulate cortex is particularly involved in social-emotional functions^29,30^ and is more damaged in bvFTD compared to other FTD subtypes^31^ . The right lingual cortex has an important role in emotional processes like visual identification of facial expressions^32^ and could be part of the neural correlates for apathy^33^.

The nfvPPA group was classified mainly on the basis of the AngleR values in the left pars opercularis. This region includes Broca’s Area for motor language function and has a central role in distinguishing nfvPPA from other groups, consistent with previous studies^34,35^.

Other key regions used to classify the FTD subgroups were the right caudal and rostral middle frontal cortices. As shown by previous studies, these regions are important for executive functions^36^ and are usually involved in FTD progression^37^.

Finally, in line with the recent literature of motor dysfunction in FTD patients^38^, bilateral precentral cortex changes were found in all patient groups.

The main limitation of the present study is the modest sample size of all cohorts. The small sample size could have an effect on feature selection and the classification power. Future research on a larger cohort will help to further advance and support the findings. Additional measures such as assessment of tau protein quantification using CSF or PET markers could also be useful.

In conclusion, we suggest that cortical diffusion measures are promising non-invasive neuroimaging features that could be help to support the diagnosis of FTD and FTD subtypes. With further validation as FTD subtype biomarkers, these cortical measurements, could help to identify the characteristics of vulnerable brain regions to be targeted for new drug treatments.

## Supplementary information

Supplementary information

## Data Availability

The data that support the findings of this study are available from the corresponding author upon reasonable request.

## Abbreviations

bvFTD: Behavioural variant of frontotemporal dementia
svPPA: Semantic variant primary progressive aphasia
nfvPPA: Non-fluent/agrammatic variant primary progressive aphasia
AngleR: Angle between the principal diffusion direction and the radial minicolumn direction within the cortex
PerpPD: The component of the principal diffusion vector that was perpendicular to the radial minicolumn direction within the cortex
ParlPD: The component of the principal diffusion vector that was parallel to the radial minicolumn direction within the cortex
GM: Grey matter
MD: Mean diffusivity
