# Supplementary material for: Using diffusion tensor imaging to detect cortical changes in fronto-temporal dementia subtypes
Source: Sci Rep. 2020 Jul 8;10:11237. doi: 10.1038/s41598-020-68118-8 (PMC7343779; doi:10.1038/s41598-020-68118-8)
Supplement: Supplementary file 1 — Supplementary information [file 41598_2020_68118_MOESM1_ESM.docx]

Title: **Using Diffusion Tensor Imaging to detect cortical changes in Fronto-Temporal Dementia subtypes**

Running head: Cortical MRI analysis in Fronto-Temporal Dementia

Torso M^1,2^ PhD, Bozzali M^3,4^ MD, Cercignani M^5^ PhD, Jenkinson M^6^ PhD, Chance SA^1,2^ DPhil and frontotemporal lobar degeneration neuroimaging initiative (FTLDNI).

1 Nuffield Department of Clinical Neurosciences, University of Oxford, Oxford, UK

2 Oxford Brain Diagnostics, Oxford Centre for Innovation, New Road, Oxford, OX1 1BY, UK

3 Neuroimaging Laboratory, Santa Lucia Foundation, Rome, Italy

4 ‘Rita Levi Montalcini’ Department of Neuroscience, University of Turin, Turin, Italy

5 Clinical Imaging Sciences Centre, Department of Neuroscience, Brighton and Sussex Medical School, University of Sussex, Brighton, UK

6 Wellcome Centre for Integrative Neuroimaging, FMRIB, Nuffield Department of Clinical Neurosciences, University of Oxford, Oxford, UK

**Supplemental Material**

**Table 1 Cortical and subcortical volumetrics**

|  | **Selection Cohort** | | | |  | **Training Cohort** | | | |  | **Test Cohort** | | | |
| --- | --- | --- | --- | --- | --- | --- | --- | --- | --- | --- | --- | --- | --- | --- |
| fr= Volume/ ICV | **HS** | **bvFTD** | **svPPA** | **nfvPPA** |  | **HS** | **bvFTD** | **svPPA** | **nfvPPA** |  | **HS** | **bvFTD** | **svPPA** | **nfvPPA** |
| Cortical GM fr | 0.263 (0.028) | 0.241*  (0.029) | 0.236*#  (0.031) | 0.243*  (0.027) |  | 0.267 (0.035) | 0.236*  (0.020) | 0.228*#  (0.026) | 0.237*  (0.029) |  | 0.272 (0.047) | 0.246*  (0.050) | 0.248*#  (0.026) | 0.258*  (0.029) |
| Subcortical GM fr | 0.037  (0.003) | 0.031*  (0.002) | 0.029*  (0.003) | 0.030*  (0.003) |  | 0.035  (0.003) | 0.030*  (0.002) | 0.029*  (0.004) | 0.028*  (0.003) |  | 0.036  (0.002) | 0.028*  (0.004) | 0.030*  (0.003) | 0.031*  (0.004) |
| WM fr | 0.153  (0.022) | 0.142*  (0.036) | 0.138*  (0.041) | 0.136*  (0.032) |  | 0.160  (0.029) | 0.145*  (0.036) | 0.139*  (0.041) | 0.141*  (0.032) |  | 0.156  (0.010) | 0.137*  (0.016) | 0.133*  (0.012) | 0.130*  (0.013) |
| WMHs fr | 0.00002  (0.00002) | 0.00003  (0.00003) | 0.00003  (0.00002) | 0.00002  (0.00002) |  | 0.00002  (0.00003) | 0.00002  (0.00005) | 0.00003  (0.00003) | 0.00002  (0.00002) |  | 0.00001  (0.00001) | 0.00002  (0.00002) | 0.00002  (0.00003) | 0.00001  (0.00002) |
| L Hippocampal fr | 0.005  (0.0006) | 0.004*  (0.0004) | 0.004*  (0.0003) | 0.004  (0.0002) |  | 0.005  (0.0002) | 0.004*  (0.0006) | 0.004*  (0.0006) | 0.004  (0.0003) |  | 0.005  (0.0005) | 0.004*  (0.0006) | 0.004*  (0.0004) | 0.005  (0.0004) |
| R Hippocampal fr | 0.005  (0.0008) | 0.004*  (0.0003) | 0.004*  (0.0005) | 0.004  (0.0004) |  | 0.005  (0.0006) | 0.004*  (0.0005) | 0.004*  (0.0008) | 0.004  (0.0005) |  | 0.005  (0.0009) | 0.004*  (0.0008) | 0.004*  (0.0005) | 0.005  (0.0008) |
| L thalamus fr | 0.004  (0.0003) | 0.003  (0.0002) | 0.004  (0.0005) | 0.004  (0.0005) |  | 0.004  (0.0003) | 0.003  (0.0003) | 0.004  (0.0007) | 0.003  (0.0004) |  | 0.004  (0.0004) | 0.003  (0.0006) | 0.004  (0.0004) | 0.003  (0.0007) |
| R Thalamus fr | 0.004  (0.0003) | 0.004  (0.0004) | 0.004  (0.0004) | 0.003  (0.0007) |  | 0.004  (0.0005) | 0.004  (0.0002) | 0.004  (0.0005) | 0.004  (0.0005) |  | 0.004  (0.0004) | 0.004  (0.0006) | 0.004  (0.0005) | 0.004  (0.0006) |
| L Caudate fr | 0.002  (0.0003) | 0.002  (0.0002) | 0.002  (0.0004) | 0.002  (0.0002) |  | 0.002  (0.0003) | 0.002  (0.0002) | 0.002  (0.0003) | 0.002  (0.0002) |  | 0.002  (0.0003) | 0.002  (0.0004) | 0.002  (0.0005) | 0.002  (0.0004) |
| R Caudate fr | 0.002  (0.0002) | 0.002  (0.0002) | 0.002  (0.0003) | 0.002  (0.0003) |  | 0.002  (0.0003) | 0.002  (0.0003) | 0.002  (0.0003) | 0.002  (0.0001) |  | 0.002  (0.0003) | 0.002  (0.0005) | 0.002  (0.0003) | 0.002  (0.0005) |
| L Putamen fr | 0.003  (0.0003) | 0.003  (0.0004) | 0.003  (0.0004) | 0.002  (0.0003) |  | 0.003  (0.0005) | 0.003  (0.0003) | 0.003  (0.0006) | 0.002  (0.0007) |  | 0.003  (0.0004) | 0.002  (0.0005) | 0.003  (0.0004) | 0.002  (0.0005) |
| R Putamen fr | 0.003  (0.0004) | 0.002  (0.0003) | 0.002  (0.0003) | 0.002  (0.0002) |  | 0.003  (0.0006) | 0.002  (0.0003) | 0.002  (0.0002) | 0.002  (0.0001) |  | 0.002  (0.0002) | 0.002  (0.0004) | 0.002  (0.0003) | 0.002  (0.0005) |
| L Amygdala fr | 0.0007  (0.0002) | 0.0006*  (0.0003) | 0.0006*  (0.0001) | 0.0006*  (0.0002) |  | 0.0007  (0.0001) | 0.0006*  (0.0001) | 0.0006*  (0.0002) | 0.0006*  (0.0001) |  | 0.0007  (0.0001) | 0.0006*  (0.0001) | 0.0006*  (0.0002) | 0.0006*  (0.0001) |
| R Amygdala fr | 0.0009  (0.0003) | 0.0008  (0.0003) | 0.0007*  (0.0002) | 0.0007*  (0.0002) |  | 0.0008  (0.0001) | 0.0008  (0.0002) | 0.0007*  (0.0001) | 0.0007*  (0.0002) |  | 0.0010  (0.0001) | 0.0008*  (0.0002) | 0.0005*  (0.0002) | 0.0009  (0.0001) |
| L Accumbens fr | 0.0003  (0.00004) | 0.0002*  (0.00003) | 0.0002*  (0.00005) | 0.0002*  (0.00003) |  | 0.0003  (0.00006) | 0.0002*  (0.00004) | 0.0002*  (0.00009) | 0.0002*  (0.00004) |  | 0.0003  (0.00005) | 0.0002*  (0.00007) | 0.0002*  (0.00006) | 0.0002*  (0.00008) |
| R Accumbens fr | 0.0003  (0.00005) | 0.0002*  (0.00006) | 0.0002*  (0.00007) | 0.0002*  (0.00004) |  | 0.0003  (0.00007) | 0.0002*  (0.00004) | 0.0002*  (0.00008) | 0.0002*  (0.00005) |  | 0.0003  (0.00005) | 0.0002*  (0.00009) | 0.0002*  (0.00008) | 0.0003  (0.00007) |
| Brainstem fr | 0.0139  (0.001) | 0.0123*  (0.001) | 0.0127*  (0.002) | 0.0122*  (0.002) |  | 0.0135  (0.001) | 0.0120*  (0.001) | 0.0125*  (0.002) | 0.0121*  (0.002) |  | 0.0138  (0.001) | 0.0126*  (0.0011) | 0.0130  (0.002) | 0.0127*  (0.001) |
| CC fr | 0.0020  (0.0003) | 0.0016*  (0.0004) | 0.0018  (0.0004) | 0.0018  (0.0005) |  | 0.0019  (0.0004) | 0.0016*  (0.0004) | 0.0017  (0.0002) | 0.0018  (0.0002) |  | 0.0021  (0.0004) | 0.0017*  (0.0005) | 0.0020  (0.0002) | 0.0019  (0.0005) |

Table 1 shows the volumetric comparisons in all the cohorts. WM= white matter; WMH= white matter hypointensities; CC= corpus callosum. To account for subjects' head size differences, all volumes were expressed as a percentage of the total intracranial volume (ICV). All results reported were significant after false discovery rate correction (FDR <0.05). *=significantly different compared to HS; #=significantly different compared to nfvPPA.
